# Significance of serum procalcitonin in sepsis

**DOI:** 10.4103/0972-5229.78214

**Published:** 2011

**Authors:** Uchil Sudhir, Ravi Kumar Venkatachalaiah, Thimmaiah Anil Kumar, Medha Yogesh Rao, Punith Kempegowda

**Affiliations:** **From:** Department of Medicine, M S Ramaiah Hospitals, MSRIT Post, New BEL Road, Bangalore, India

**Keywords:** Procalcitonin, sepsis, septic shock, severe sepsis, sepsis-related organ failure assessment score

## Abstract

**Context:**

Rapid treatment of sepsis is of crucial importance for survival of patients. Specific and rapid markers of bacterial infection have been sought for early diagnosis of sepsis. One such measurement, Procalcitonin (PCT), has recently become of interest as a possible marker of the systemic inflammatory response to infection.

**Aims:**

This study was done to find out the common sources of sepsis and to evaluate the diagnostic value of PCT, its predictive value and its relation with Sepsis-related Organ Failure Assessment (SOFA) scores and mortality in various stages of sepsis.

**Settings and Design:**

The prospective study was conducted at our tertiary care center from October 2006 to December 2008. A total of 100 patients were included in the study. The study sample included all patients aged above 18 years presenting consecutively to our center during the study period with acute sepsis. They were divided into three groups: sepsis, severe sepsis and septic shockbased on standardized criteria.

**Materials and Methods:**

PCT and various other relevant factors were measured in all study subjects. These parameters were compared among the three study groups. The statistical analyses were done using Student “*t*” test and two-way analysis of variance (ANOVA).

**Results:**

Respiratory tract infection was the most common source of sepsis. PCT proved to be an excellent indicator of sepsis with sensitivity of 94%. There was a significant association between serum PCT and SOFA scores (*P* < 0.05). Serum PCT levels did not predict mortality in the present study.

**Conclusions:**

PCT is among the most promising sepsis markers, capable of complementing clinical signs and routine lab parameters suggestive of severe infection.

## Introduction

Sepsis is reported to be the most common cause of death in non-coronary Intensive Care Unit (ICU). It is an increasingly common cause of mortality and morbidity particularly in elderly, Immunocompromised and critically ill patients. Approximately, 25–35% of patients with severe sepsis and 40–55% of patients with septic shock die within 30 days.[1]

Patients with systemic infection and organ dysfunction or shock are often difficult to distinguish from patients with similar clinical signs and lab finding, but without infection. The established biological markers of inflammation (leukocytes, C-reactive protein) may often be influenced by parameters other than infection and may only be slowly released during progression of an infection. Positive bacteriological results may be caused by contamination and negative results do not exclude sepsis. Since these common clinical and lab measurements lack sensitivity and specificity, other tests are needed to give an early marker of the infectious cause of a generalized inflammatory response to allow early diagnosis and for the use of specific treatment.

One such measurement, Procalcitonin (PCT), has recently become of interest as a possible marker of the systemic inflammatory response to infection. Numerous studies abroad have proved its efficacy as a marker of critical illness.[2–6] Alas no similar studies exist in Indian literature. Since the expression of serum PCT depends on the genetic constituents of the population, it is necessary to prove its efficacy as a marker of sepsis in Indian population. This study was done to find out the common sources of sepsis, severe sepsis and septic shock and to evaluate the diagnostic value of serum PCT, its predictive value and its relation with Sepsis-related Organ Failure Assessment (SOFA) scores and mortality in various stages of sepsis.

## Materials and Methods

This prospective observational study was conducted in a tertiary care center in Bangalore from October 2006 to December 2008. The study was approved by the Institution’s Ethical Committee. The study sample included all patients aged above 18 years presenting consecutively to our center during the study period with acute sepsis, as diagnosed by one of the following: clinical presentation of sepsis with positive blood culture, clinical presentation of urinary tract infection with positive urine culture, clinical presentation of pneumonia with supporting radiological features and positive sputum culture, or other conditions with clinical and laboratory features compatible with sepsis. Patients with history of malignancy, trauma or recent surgery were excluded from the study. The study subjects were grouped into sepsis, severe sepsis and septic shock based on American College of Chest Physicians/Society of Critical Care Medicine (ACCP/SCCM) Consensus guidelines.[7]

Exactly 52, 25 and 23 patients were included with the diagnosis of sepsis, severe sepsis and septic shock, respectively. Blood samples were drawn from all patients within 24 hours of admission to the ICU for complete blood count, erythrocyte sedimentation rate (ESR), prothrombin time, activated partial thromboplastin time, liver and kidney function tests, blood culture, and estimation of serum PCT. Urine routine, chest X-ray and ultrasound abdomen were done for all patients. Appropriate cultures were sent from each patient based on presentation and clinical suspicion for identifying the focus of infection. IgM antibodies against dengue and *Leptospira* and peripheral blood smear for malarial parasite were done when indicated. SOFA score was calculated in severe sepsis and septic shock groups and correlated with serum PCT.

### 

#### Serum PCT measurement

Serum PCT was measured by using PCT-Q (B.R.A.H.M.S, Berlin). It is an immunochromatografic test for the semi-quantitative detection of serum PCT. With an incubation period of only 30 minutes, the test neither depends on apparatus nor needs calibrating.[8] Based on the test sensitivity and specificity provided by the manufacturer, the subject was said to have serum PCT test positive if values were >0.5 ng/ml.

The test uses a monoclonal mouse anti-catacalcin antibody conjugated with colloidal gold (tracer) and a polyclonal sheep anti-calcitonin antibody (solid phase). After the patient sample (serum or plasma) has been applied to the test strip, the tracer binds to the serum PCT in the sample and a marked antigen antibody complex forms. This complex moves by means of capillarity through the test system and, in the process, passes through the area containing the test band. Here, the marked antigen antibody complex binds to the fixed anti-calcitonin antibodies and forms a sandwich complex. At a serum PCT concentration ≥0.5 ng/ml, this sandwich complex can be seen as a reddish band. The color intensity of the band is directly proportional to the serum PCT concentration of the sample.

#### Statistical analysis

Statistical analyses were performed using Statistical Package for Social Survey (SPSS) for Windows version 16.0. Student *“t”* test was used to find the strength of association between serum PCT and SOFA scores; and serum PCT and mortality. Two-way analysis of variance (ANOVA) was applied to calculate the association between the serum PCT level and grade of sepsis. Two-tailed *“P”* values below 0.05 were considered significant. The results was tabulated and graphically represented using Microsoft Office for Windows 2007.

## Results

**Figure 1:**
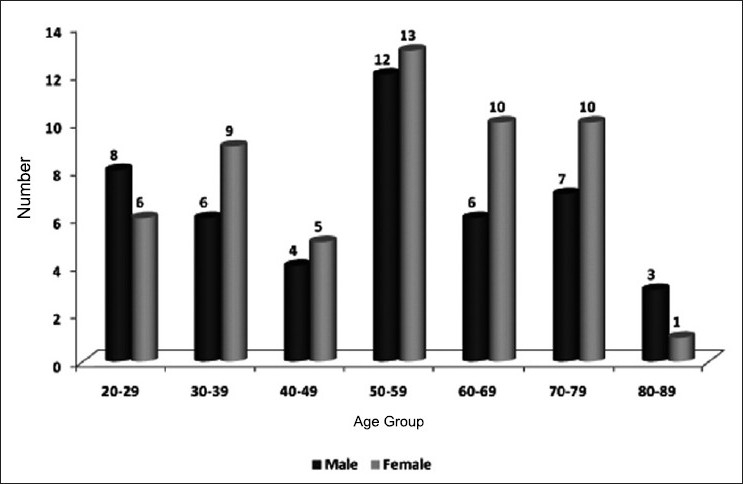
Age- and gender-wise distribution of the study subjects

Overall, 100 patients were included in the study from a total 886 screened for the inclusion and exclusion criteria during the study period who were divided into three groups- sepsis (52), severe sepsis (25) and septic shock (23)- as per the study guidelines.. The age-wise and gender-wise distribution of the study subjects is given in Figure 1. In our study, the mean age of the study population was 52.5 years.

**Figure 2:**
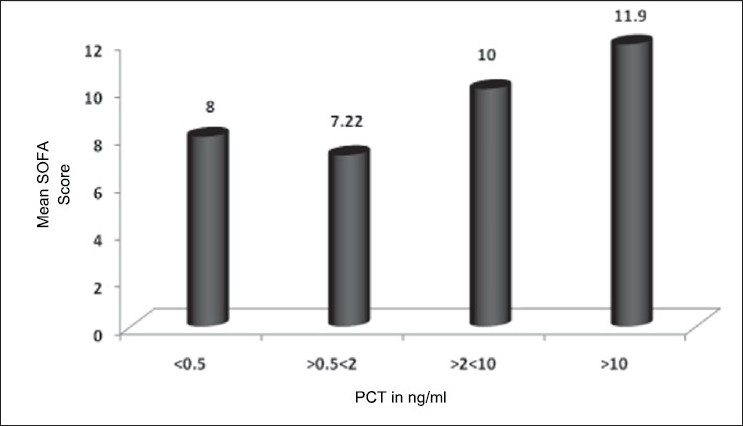
Comparison of serum PCT and mean SOFA score in severe sepsis and septic shock

**Figure 3:**
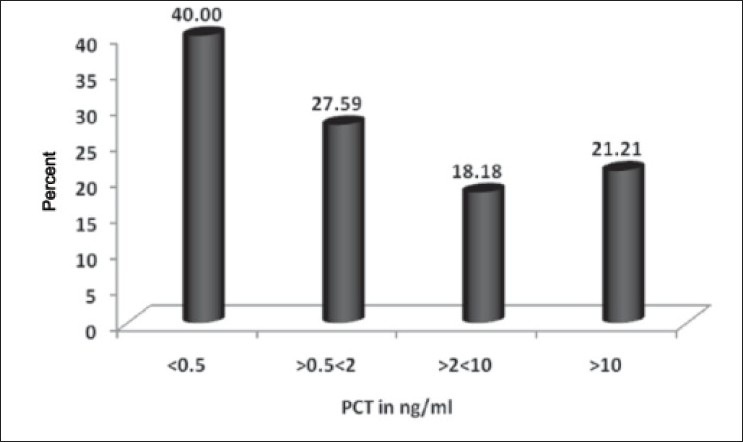
Relation between serum PCT with mortality in sepsis, severe sepsis and septic shock

The various sites of the primary source of infection are given in Table 1. Serum PCT was positive in 94% of the study subjects [Table 2]. There was no significant association between the level of serum PCT and grade of sepsis (*P* > 0.05). The mean SOFA scores in sepsis, severe sepsis and septic shock were 1.96, 9.68 and 8.52, respectively. Higher SOFA score levels were associated with significantly higher serum PCT concentrations (*P* < 0.05) [Figure 2]. There was no significant association between the level of serum PCT at presentation and mortality in the present study (*P* > 0.05) [Figure 3].

**Table 1:** Primary source of infection in sepsis

Source of infection	Sepsis	Severe sepsis	Septic shock	Total
Respiratory tract	28	3	9	40
Urinary tract	12	1	6	19
GIT	5	0	3	8
Malaria	1	2	0	3
Leptospiroses	1	4	0	5
Dengue	0	2	1	3
Cellulites	1	0	1	2
Source not found	4	13	3	20

**Table 2:** Serum procalcitonin in sepsis, severe sepsis and septic shock patients

Diagnosis	Serum PCT range (ng/ml)				
	<0.5	>0.5 and <2	>2 and <10	>10	Total
Sepsis	4 (7.7)	15 (28.8)	19 (36.5)	14 (26.9)	52 (100.0)
Severe sepsis	1 (4.0)	5 (20.0)	9 (36.0)	10 (40.0)	25 (100.0)
Septic shock	1 (4.3)	4 (17.4)	7 (30.4)	11 (47.8)	23 (100.0)

Values in parenthesis are in percentage

## Discussion

Our study population was a large, diverse group of critically ill adult patients with sepsis, admitted to the medical ICU. It was designed as a real-life study, to closely resemble clinical practice. We evaluated the combined role of serum PCT and other clinical indicators of inflammation as predictors of sepsis in which we explored the diagnostic accuracy of these different parameters from a clinical perspective.

Incidence was more in patients aged over 50 years (60%). The age distribution is similar to studies done around the world. A western study reported a higher incidence of sepsis in patients aged above 57 years.[9] The mean age in an epidemiological study of sepsis in India was 54.9 years.[10]

We found a slightly higher percentage of males affected with sepsis compared to females in the present study. Studies by previous workers also indicated a higher incidence among men. Martin *et al*.[9] studied the demography, temporal incidence and changes in incidence and outcome of sepsis over 20 years in the United States reported that sepsis was more common in men, accounting for 48.1% of cases on average per year and men were more likely to have sepsis than women with a mean annual relative risk of 1.28. Todi and group reported from a multicenter trial done at 12 centers in India that sepsis was more common in males.[10]

Respiratory tract infection (40%) was the most common source of sepsis in our study [Table 1]. 27 cases had lobar pneumonia and 12 cases had bronchopneumonia. *Streptococcus pneumoniae* was the most common agent causing community acquired pneumonia in the present study. In 20% of patients, the primary source could not be identified. Urinary tract infection was the second most common focus which may be partly due to more number of elderly patients with risk factors like diabetes. According to Calandra *et al*.,[11] six common infection sites identified in the causation of sepsis were pneumonia, blood stream infections including infective endocarditis, intravascular catheter related sepsis, intra-abdominal infections, urosepsis and surgical wound infections.

Dengue was found to be primary source of sepsis in three cases. Studies have shown a mild elevation of serum PCT in viral infections.[12] In contrast, two of three patients in our study had mild elevation of serum PCT and one had marked elevation of serum PCT level. Malaria was the underlying cause of sepsis in three cases. Mild elevation of serum PCT in malaria has been reported in literature.[13,14]

Serum PCT has 94% sensitivity in the present study. Harbarth *et al*. reported a sensitivity of 97%.[15] The present results confirm earlier findings that demonstrate serum PCT as among the most promising sepsis markers in critically ill patients, capable of complementing clinical signs and routine lab parameters suggestive of severe infection at the time of ICU admission. Brunkhorst *et al*. reported from their study that serum PCT levels increase with the increasing severity of the inflammatory response to infection.[6] In contrast, we did not find any association between the serum PCT level and the diagnosis. These results are in agreement with the reports of Suprin and colleagues.[16]

SOFA score was calculated in 48 patients (severe sepsis and septic shock) in the present study. It was found that higher SOFA score levels were associated with significantly higher serum PCT plasma concentrations. Similar results have been found in various studies worldwide.[17–19]

Mortality was seen in 23 patients (23%) in the present study. A study by Martin *et al*.[9] showed that mortality in patients with sepsis from various centers varied between 16.8 and 31.8%. Sands *et al*.[20] who studied sepsis in eight academic medical centers reported a mortality rate of 34%. Mortality could be attributed to age and various risk factors that are more common in that age group. An additional risk factor for increased mortality would be diabetes.

Serum PCT, normally produced in the C-cells of the thyroid gland, is the precursor of calcitonin. A specific protease cleaves serum PCT to calcitonin, catacalcin, and an N-terminal residue. Normally, all serum PCT is cleaved and none is released into the blood stream. Serum PCT levels are therefore undetectable (<0.1 ng/ml) in healthy humans. During severe infections with systemic manifestations, however, serum PCT levels may increase to over 100 ng/ml. In these conditions, serum PCT is probably produced by extra-thyroid tissues. Patients who have previously undergone total thyroidectomy still produce high levels of serum PCT during severe infection. The exact origin of serum PCT during sepsis is uncertain. The (patho) physiological role of serum PCT during sepsis is not clear.[3,21]

Serum PCT levels increase during severe generalized bacterial, parasitic or fungal infections with systemic manifestation. In severe viral infections, or inflammatory reactions of non-infectious origin, serum PCT levels do not increase or only show a moderate increase. Compared to the relatively short half-lives of cytokines such as tumor necrosis factor (TNF)-a and interleukin (IL)-6, the half-life of serum PCT in the systemic circulation is 25-30 hours rather long.[22] Because of these properties, serum PCT has been proposed as an indicator of severe generalized infections or sepsis.[4,5,23]

Serum PCT is not a marker of infection as such since localized infections or infections with no systemic manifestation cause a limited, if any, increase in serum PCT levels. Although elevated serum PCT values during severe infections may decrease to very low levels with appropriate therapy, this does not always indicate complete eradication of the infection but only that generalization of the infection or the systemic response is under control.[24]

Systemic inflammatory syndrome of non-infectious etiologies also leads to increases in serum PCT levels. Patients after major trauma or surgery and patients after cardiopulmonary bypass may present with increased serum PCT levels without any evidence of severe infection. However, the median values under these conditions are usually lesser than those found during severe sepsis and septic shock.[6]

Our study has several important implications for clinicians. Although the present study population is small to commit to the importance of serum PCT in critical care, it definitely indicates that serum PCT may be included in the battery of infections to help in the management of sepsis in critical care. First, as a new test to diagnose sepsis on ICU admission, serum PCT offers a high level of accuracy that other currently available tests cannot provide. Although the accuracy of serum PCT reference range is not perfect, it may guide physicians in their clinical decision making and their stepwise approach to the complex management of critically ill patients with sepsis requiring several interventions in a short period of time. The test can be performed within 30 minutes and gives valuable information long before culture results are available.

In summary, incidence of sepsis was higher in patients aged >50 years and males. Respiratory tract infection was the most common source of sepsis. Serum PCT proved to be an excellent indicator of sepsis in critically ill patients, with sensitivity of 94%. Our results indicate that clinical variables are of modest diagnostic value for the diagnosis of sepsis on ICU admission.

## Conclusion

The present study demonstrates serum PCT to be among the most promising sepsis markers in critically ill patients, capable of complementing clinical signs and routine lab parameters suggestive of severe infection at the time of ICU admission. Serum PCT measurement appears to be a better predictor to distinguish patients with sepsis and patients without sepsis when compared to blood cell counts or body temperature or ESR. Thus, our data raise the possibility that the addition of serum PCT to the standard work-up of critically ill patients with suspected sepsis could increase diagnostic certainty and improve patient management.
